# Association of the oxytocin receptor gene with attitudinal trust: role of amygdala volume

**DOI:** 10.1093/scan/nsy075

**Published:** 2018-09-07

**Authors:** Kuniyuki Nishina, Haruto Takagishi, A S R Fermin, Miho Inoue-Murayama, Hidehiko Takahashi, Masamichi Sakagami, Toshio Yamagishi

**Affiliations:** 1 Graduate School of Brain Sciences, Tamagawa University, Tamagawagakuen, Machida-shi, Tokyo, Japan; 2 Brain Science Institute, Tamagawa University, Tamagawagakuen, Machida-shi, Tokyo, Japan; 3 Wildlife Research Center, Kyoto University, Tanaka-Sekiden-cho, Sakyo-ku, Kyoto, Japan; 4 Graduate School of Medicine, Kyoto University, Shogoin-Kawaracho, Sakyo-ku, Kyoto, Japan; 5 Graduate School of International Corporate Strategy, Hitotsubashi University, Hitotsubashi, Chiyoda-ku, Tokyo, Japan

**Keywords:** oxytocin receptor gene, single nucleotide polymorphism, amygdala, attitudinal trust

## Abstract

Previous studies have shown that genetic variations in rs53576, a common variant of the oxytocin receptor gene (*OXTR*) resulting from a single nucleotide polymorphism involving an adenine (A)/guanine (G) transition, are associated with attitudinal trust in men. However, the pathway from gene to behaviour has not been elucidated. We conducted the present study to determine whether amygdala volume mediates the association between *OXTR* rs53576 genotypes and attitudinal trust. Our results revealed that the left amygdala volume was significantly smaller in GG men than in AA and AG men, whereas it was significantly smaller in AA and AG women than in GG women. In addition, the left amygdala volume was negatively associated with attitudinal trust in men, whereas there was no such association in women. We also found a significant mediation effect of the left amygdala volume on the association between *OXTR* rs53576 genotypes and attitudinal trust in men. The results of our study suggest that the left amygdala volume plays a pivotal role in the association between *OXTR* rs53576 genotypes and attitudinal trust in men.

## Introduction

Trust plays a pivotal role not only in interpersonal relationships, but also in the world of economics, politics, and law; moreover, it has attracted a great deal of attention in the field of social sciences (Barber, 1983; Putnam, 1993; Knack and Keefer, 1997; Yamagishi, 2011; Uslaner and Rothstein, 2005). With regard to interpersonal relationships, trust provides opportunities to form beneficial relationships and prospects for social exchange. Because trust is a widespread psychological trait found acrosssocieties and the heritability of trust is high (Casarini *et al*., 2008), trust may have a biological basis. Accordingly, recent studies have examined the biological foundation of human trust and found that oxytocin modulates trust behaviour (Kosfeld *et al*., 2005; Baumgartner *et al*., 2008; Zhong *et al*., 2012).

Oxytocin is a neuropeptide produced in the paraventricular hypothalamic nucleus and, as a neurotransmitter in the central nervous system, it affects various aspects of human sociality (Donaldson and Young, 2008; Meyer-Lindenberg *et al*., 2011). A previous study (Kosfeld *et al*., 2005) found that the intranasal administration of oxytocin enhanced trust behaviour in a trust game (Berg *et al*., 1995). Given the possibility of trust betrayal by others, trust behaviours are considered to reflect risk-taking decisions in social exchange settings (Bohnet and Zeckhauser, 2004; Kosfeld *et al*., 2005; Yamagishi, 2011; Yamagishi *et al*., 2015). Baumgartner *et al*. (2008) found that amygdala activation was attenuated by oxytocin when the participants decided whether to trust opponents with the feedback that half of the trust behaviour was betrayed from the opponents in the previous rounds. They argued that oxytocin inhibits aversion to social risk by attenuating the function of the amygdala. However, as a more recent study argued that oxytocin has no effect on trust (Nave *et al*., 2015), the association between oxytocin and trust has attracted more attention in various fields, but needs further research to be fully understood.

**Fig. 1:**
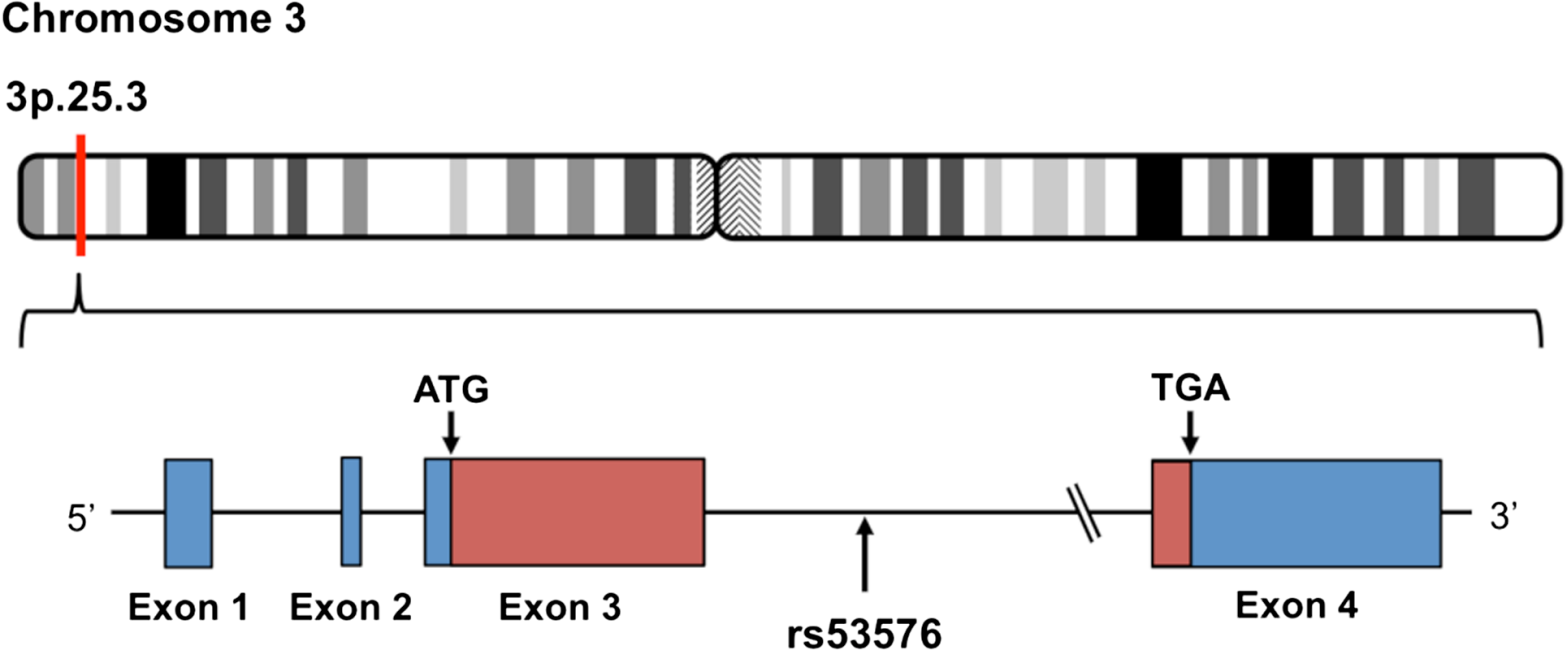
Location of the oxytocin receptor gene (*OXTR*) rs53576. rs53576 is a single nucleotide polymorphism in the third intron on the *OXTR*.

Further evidence supporting the role of oxytocin in trust is provided by genetic studies (Apicella *et al*., 2010; Krueger *et al*., 2012; Nishina *et al*., 2015). The oxytocin receptor (*OXTR*) gene is localised to the human chromosome 3p25.3 and has four exons (Inoue *et al*., 1994; Figure 1). *OXTR* encodes the G protein related to the oxytocin receptor, which regulates anxiety, bonding and maternal behaviour (Veenema and Neumann, 2008). Krueger *et al*. (2012) found that genetic variations in *OXTR* rs53576, a single nucleotide polymorphism in *OXTR* involving an adenine (A)/guanine (G) transition, were associated with trust behaviour in young men. They showed that men homozygous for the G allele (GG genotype) showed higher levels of trust in a trust game compared with A allele carriers (AG or AA genotype). However, in another study, Apicella *et al*. (2010) did not find an association between *OXTR* rs53576 genotypes and trust behaviour in men or women.

The sex ratio of participants was biased in previous studies: the ratio of men was 100% (Krueger *et al*., 2012) and about 20% (Apicella *et al*., 2010). Consequently, we conducted an experiment for men and women from a wide range of ages (20s–50s) with a sex ratio that was almost even (Nishina *et al*., 2015). We found that the levels of behavioural and attitudinal trust in men homozygous for the G allele were significantly higher than the levels in A allele carriers (AA/AG), and that attitudinal trust mediates the association between *OXTR* rs53576 genotypes and behavioural trust in men (Nishina *et al*., 2015). These findings indicate that *OXTR* rs53576 genotypes are directly associated with attitude, and not behaviour, in men and is associated with behavioural trust via changes in attitudinal trust.

Although previous studies (Krueger *et al*., 2012; Nishina *et al*., 2015) showed that *OXTR* rs53576 genotypes are associated with trust, the pathway from genotype to phenotype has not been elucidated. To understand the biological mechanism of trust, the relationship between genes and trust must be investigated. To date, two imaging genetics studies have examined the association between *OXTR* rs53576 genotypes and the brain structure in young adults (Tost *et al*., 2010; Wang *et al*., 2014). Tost *et al*. (2010) found that men with a GG genotype have a smaller amygdala volume compared with men with AG or AA genotypes, whereas women with a GG genotype have a larger amygdala volume compared with women with AG or AA genotypes. In comparison, Wang *et al*. (2014) found that women with GG and AG genotypes have larger amygdala volumes compared with women with the AA genotype. Indeed, previous studies showed that the oxytocin receptor is abundant in the amygdala (Insel and Shapiro, 1992; Febo *et al*., 2005; Ophir *et al*., 2012) and that intranasal oxytocin administration can inhibit the function of the amygdala when individuals face untrustworthy opponents (Baumgartner *et al*., 2008). These results suggest the possibility that *OXTR* rs53576 variations are associated with the amount of oxytocin receptors in the amygdala, consequently affecting amygdala function. In other words, it is considered that men with the GG genotype on *OXTR* rs53576 display higher levels of attitudinal trust because aversion to social risk is inhibited by oxytocin.

Although previous studies have demonstrated an association between the *OXTR* rs53576 genotypes and amygdala volume, this association was not examined with respect to trust; examining this relationship will enhance our understanding of the biological mechanism of trust. In the present study, we analysed three aspects concerning the relationship between the *OXTR* rs53576 genotypes, amygdala volume and attitudinal trust. First, we examined whether genetic variations in *OXTR* rs53576 are associated with amygdala volume; although the association is already shown in young adults (Tost *et al*., 2010; Wang *et al*., 2014), we investigated whether this association is observed in individuals belonging to a wider age range, from 20–59 years of age. Second, we examined whether amygdala volume is associated with the level of attitudinal trust. According to studies on social anxiety, characterised by avoidance behaviour in interpersonal relationships, individuals with high levels of social anxiety have a large amygdala volume and a high level of amygdala activation (Tillfors *et al*., 2001, 2002; Machado-de-Sousa *et al*., 2014). Because anxiety in social exchange settings is negatively associated with attitudinal trust (Yamagishi, 2011), we predicted that the amygdala volume is negatively associated with attitudinal trust levels. Finally, we examined whether amygdala volume mediates the association between *OXTR* rs53576 genotypes and attitudinal trust in men. To investigate all three associations, we used voxel-based morphometry, which is widely used in neuroanatomical studies to measure individual differences in the brain structure (Kanai and Rees, 2011). Our analyses incorporated MRI data and information reported in our previous study (Nishina *et al*., 2015) that examined the association between *OXTR* rs53576 genotypes and behavioural and attitudinal trust. However, this is the first study on the relationships among *OXTR* rs53576 genotypes, the brain structure and attitudinal trust.

## Materials and Methods

### Participants

Six hundred nonstudent residents living in a moderately wealthy suburb of Tokyo were selected from a list of 1670 applicants who responded to a brochure distributed to approximately 180 000 households. These 600 individuals included 75 men and 75 women in each 10-year age group from 20–59 years. The study was conducted in eight phases over 65 months. Each phase lasted three to six hours, during which various economic games and cognitive experiments were conducted and psychological tests were administered. Findings concerning some of the data collected during the eight phases have been previously reported (Yamagishi et al., 2014; Nishina *et al*., 2015; Yamagishi et al., 2015; Matsumoto *et al*., 2016; Yamagishi et al., 2016a, 2016b). The participants answered questions related to attitudinal trust during the first (17 May 2012–22 July 2012) and seventh(25 November 2014–25 January 2015) phases. We collected magnetic resonance imaging (MRI) data during the second phase (6 November 2012–16 February 2013) and buccal cells for DNA extraction were collected during the seventh phase. MRI and genetic data were available for a total of 410 participants: 211 men and 199 women; we focused on these participants in the following analyses. We collected education and annual income information as well as a subjective social class rating that describes the perception of the participants regarding their involvement in society. Demographic data for the 410 participants are shown in Figures S1, S2, S3 and S4.

All experimental protocols were approved by the Ethics Committee of Tamagawa University, where the study was conducted, and the Ethics Committee of Kyoto University Graduate School and Faculty of Medicine, where the genotyping analysis was conducted. The study conformed to the tenets of the Declaration of Helsinki and all participants provided written informed consent.

### Attitudinal trust evaluation

Participants were asked to answer the following question: ‘Do you think most people would try to take advantage of you if they got a chance, or not?’ This question is used in large-scale surveys such as the General Social Survey and the World Values Survey as well as in a previous trust study (Nishina *et al*., 2015). The question was answered in a binary form, where 0 indicated lack of trust and 1 indicated trust.

### Genotyping

We collected buccal cells from participants and preserved them in 90% ethanol until DNA extraction was performed in the seventh phase. DNA was extracted using the DNeasy Blood & Tissue Kit (QIAGEN, Tokyo, Japan) according to the manufacturer’s instructions. Genotyping of *OXTR* rs53576 was conducted using LAMP Genotyping Series Human *OXTR* (rs53576; Nippon Gene, Toyama, Japan) by mixing fluorescently labelled LAMP primers and Bst DNA polymerase. The fluorescence level of the reactant was measured by the LAMP-FLP method using a Genie II (Nippon Gene). The same protocol was used in our previous study (Nishina *et al*., 2015).

### Magnetic resonance imaging

The 3-T Siemens Trio A Tim MRI scanner at Tamagawa University Brain Science Institute was used to obtain MRI data. High-resolution anatomical images were acquired using a T1-weighted 3D magnetisation prepared rapid acquisition gradient echo (MPRAGE) sequence [repetition time (TR), 2000 ms; echo time, 1.98 ms; field of view, 256 × 256 mm; number of slices, 192; voxel size, 1 × 1 × 1 mm; average, 3 times].

### Magnetic resonance imaging data analysis

The T1-weighted images were processed and analysed using the Computational Anatomy Toolbox (CAT12, http://dbm.neuro.uni-jena.de/cat/) and Statistical Parametric Mapping software (SPM12, http://www.fil.ion.ucl.ac.uk/spm). The images were corrected for bias, marked for tissue or fluid type (grey matter, GM; white matter, WM; cerebrospinal fluid, CSF), and registered using linear (12 parameter affine) and nonlinear (warping) transformations within the CAT12 default pre-processing pipeline. This initial step generated modulated normalised images that were smoothened via the standard SPM12 smooth pipeline with a full-width at half maximum smoothing kernel of 8 × 8 × 8 mm. CAT12 was used to estimate the overall volume of GM, WM and CSF.

The pre-processed, smoothened GM images were analysed with SPM12 to ascertain brain regions where the GM density was associated with *OXTR* rs53576 genotypes. Because our previous study did not find a significant difference in attitudinal trust between the AA and AG genotype groups (Nishina *et al*., 2015), we combined the brain images for participants with these two genotypes into a single group (AA and AG group) and compared them with images for the GG genotype group. We performed a two-way analysis of variance with two dummy variables as independent variables: *OXTR* rs53576 genotypes (AA and AG = 0, GG = 1) and sex (women = 0, men = 1). The ages and total GM volume of the participants were included in the analysis as covariates of no interest to regress out any effects attributable to them.

Based on our previous hypothesis that the amygdala volume is associated with *OXTR* rs53576 genotypes, we conducted whole-brain analyses using a statistical threshold of P_FWE-SVC_ < 0.05 [family-wise error (FWE) corrected with small volume correction (SVC)] with an anatomical mask image of the amygdala (bilateral), which was constructed from the Automated Anatomical Labeling (AAL) atlas in the Wake Forest University PickAtlas software (Maldjian *et al*., 2003).

## Results

### Genotype distribution

The genotype distribution among the 410 participants was as follows: AA, *N* = 166 (40.5%); AG, *N* = 193 (47.1%); and GG, *N* = 51 (12.4%). There was no significant difference in the genotype distribution between men and women [*χ*^2^ (2) = 3.05, *P* = 0.218]. Moreover, genotype distribution was not associated with generation [*χ*^2^ (6) = 1.68, *P* = 0.946], education level [*χ*^2^ (2) = 1.31, *P* = 0.518], annual income [*χ*^2^ (12) = 8.70, *P* = 0.728] or subjective social class [*χ*^2^ (8) = 6.44, *P* = 0.598]. The demographics and genotype distribution by sex and generation are summarised in Tables S1,S2 and S3.

### Voxel-based morphometry findings

**Fig. 2:**
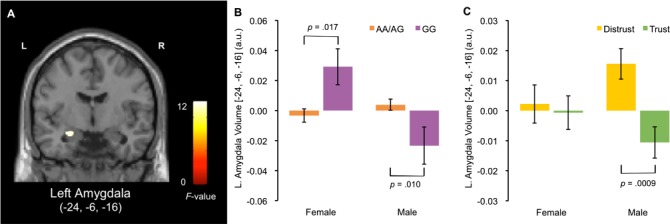
Results of voxel-based morphometry analysis conducted to assess the role of amygdala volume in the association between genetic variations in oxytocin receptor gene (*OXTR*) rs53576 and attitudinal trust. Interaction effect between *OXTR* rs53576 genotypes and sex (A). Mean volume of the left amygdala in men and women with each genotype (B). Mean volume of the left amygdala according to the participants’ levels of attitudinal trust measured in the first phase by sex. Participants were asked `Do you think most people would try to take advantage of you if they got a chance, or not?' Participants who answered NO to the question were assigned to the distrust group, while those who answered YES were assigned to the trust group. The *P*-values indicate the results of logistic regression analysis (C). Error bars show standard errors.

Whole-brain analysis revealed that the left amygdala volume showed a significant interaction effect between *OXTR* rs53576 genotypes and sex (peak coordinate: x = −24, y = −6, z = −16, *F* = 12.5, *z* = 3.31, *p*_FWE-SVC_ = 0.028, cluster size = 54; Figure 2A). The volume of the peak voxel of the left amygdala, after adjustment for age and total GM volume, was extracted and further analysed by sex. The results revealed that the left amygdala volume was significantly smaller in GG men than in carriers of A alleles [*t* (209) = 2.60, *P* = 0.01, *d* = 0.46], whereas it was significantly smaller in women carrying A alleles than in those homozygous for the G allele [*t* (197) = 2.42, *P* = 0.017, *d* = 0.57; Figure 2B]. No other brain regions showed a significant relationship between *OXTR* rs53576 genotypes and sex (Table 1).

**Table 1:** Whole-brain analysis of the association between oxytocin receptor gene (*OXTR*) rs53576 genotypes and GM volume (voxels survived at *P* < 0.001 uncorrected with extent more than 30 voxels)

Anatomical location	Peak MNI coordinates			Cluster size	*F-*value	*Z*-value
	*x*	*y*	*z*			
Interaction effect between *OXTR* rs53576 genotypes and sex						
Left amygdala	−24	−6	−16	54	12.5	3.31
Main effect of *OXTR* rs53576 genotypes						
Left anterior cingulate cortex	−8	38	20	170	16.3	3.82
Right middle occipital gyrus	28	−90	3	32	13.7	3.49
Right caudate	8	15	0	72	13.6	3.48
Right transverse temporal gyrus/Right postcentral gyrus	63	−15	15	45	12.8	3.36

Abbreviations: MNI, Montreal Neurological Institute.

We also examined the main effects of *OXTR* rs53576 genotypes using whole-brain analysis (*P* < 0.001 with extent more than 30 voxels) and found that the volumes of the left anterior cingulate cortex (x = −8, y = 38, z = 20) and right middle occipital gyrus (x = 28, y = −90, z = 3) were significantly smaller in both men and women with a GG genotype than in those with AA and AG genotypes (Table 1). In addition, the volumes of the right caudate (x = 8, y = 15, z = 0) and right transverse temporal gyrus/right postcentral gyrus (x = 63, y = −15, z = 15) were significantly larger in both men and women with a GG genotype than in those with AA and AG genotypes.

### Association between the left amygdala volume and attitudinal trust

Using the extracted volume of the peak voxel of the left amygdala, we examined whether the left amygdala volume is associated with attitudinal trust. Because 14 out of 410 participants (male = 5, female = 9) failed to answer the question concerning attitudinal trust during phases 1 and 7, we excluded those participants from the analyses. We conducted logistic regression analyses for attitudinal trust data collected during both phases using three independent variables: sex (dummy variable: male = 1), the left amygdala volume and the interaction term of sex and the left amygdala volume. For data collected in the first phase, we found a significant interaction effect between sex and the left amygdala volume (*β* = 0.13, Wald *χ*^2^ = 5.19, *P* = 0.023). A separate analysis of sex revealed that the effect of the left amygdala volume on attitudinal trust was significant in men [*β* = −0.29, Wald *χ*^2^ = 10.96, *P* = 0.0009, odds ratio < 0.001, 95% confidence interval (<0.001–0.019)], but not in women [*β* = −0.028, Wald *χ*^2^ = 0.12, *P* = 0.733, odds ratio = 0.418, 95% confidence interval (0.003–62.36)]. For the second measurement of attitudinal trust during the seventh phase, we did not find a significant interaction effect between sex and the left amygdala volume (*β* = 0.11, Wald *χ*^2^ = 3.71, *P* = 0.054, Figure S5).

**Fig. 3:**
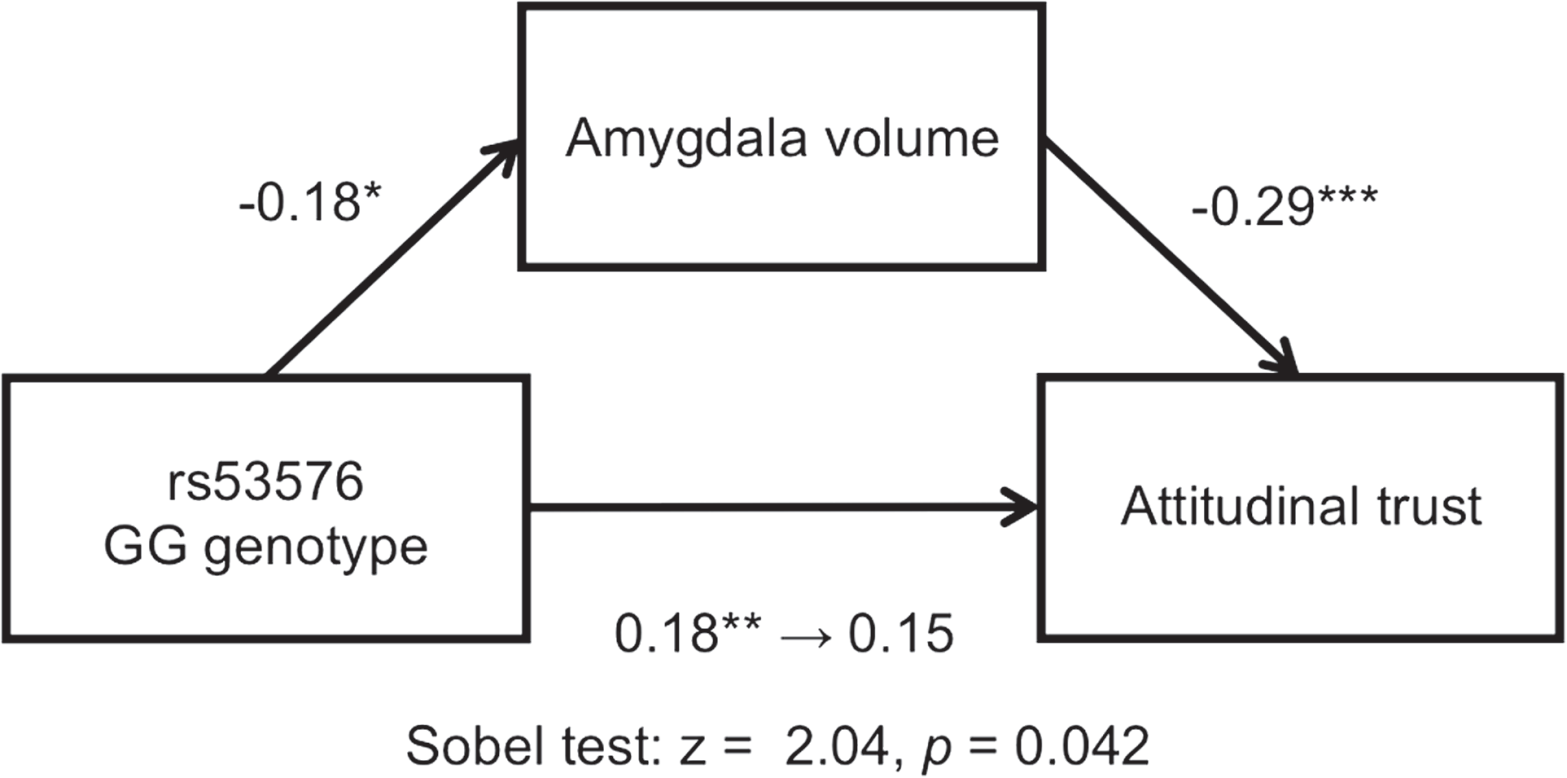
Results of mediation analysis conducted to assess the role of left amygdala volume in the association between variations in oxytocin receptor gene (*OXTR*) rs53576 and attitudinal trust in men. The left amygdala volume mediates the association between *OXTR* rs53576 (GG = 1) and attitudinal trust. ^*^P < 0.05, ^**^P < 0.01, ^***^P < 0.001.

### Mediation analysis

We conducted mediation analyses to examine whether the left amygdala volume affects the association between *OXTR* rs53576 genotypes and attitudinal trust measured in men during the first phase. Initially, we found a significant effect of the GG genotype on attitudinal trust [*β* = 0.18, Wald *χ*^2^ = 4.30, *P* = 0.038, odds ratio = 0.386, 95% confidence interval (0.157–0.949)]; however, the effect was no longer significant after controlling for the left amygdala volume [*β* = 0.15, Wald *χ*^2^ = 2.72, *P* = 0.099, odds ratio = 0.459, 95% confidence interval (0.182–1.158); Figure 3]. In addition, we found that the left amygdala volume had a significant mediation effect on the association between the GG genotype and attitudinal trust in men (Sobel test; *Z* = 2.04, *P* = 0.042).

## Discussion

We examined the associations among *OXTR* rs53576 genotypes, amygdala volume and attitudinal trust; this is the first study to show the association between *OXTR* rs53576 genotypes, amygdala volume and attitudinal trust in people of a wide age range. Our analyses revealed three important findings. First, *OXTR* rs53576 genotypes were associated with the left amygdala volume, although the direction of the association was dependent on the sex of the individual. Men with a GG genotype exhibited smaller left amygdala volumes compared with men with AA and AG genotypes, whereas women with a GG genotype exhibited larger left amygdala volumes compared with women with AA and AG genotypes. These findings indicate that oxytocin receptors affect the structure of the amygdala. Our data support some of the findings from previous studies (Tost *et al*., 2010; Wang *et al*., 2014). Tost *et al*. (2010) found an association between *OXTR* rs53576 genotypes and right amygdala volume in both men and women, while Wang *et al*. (2014) found an association between genotypes and amygdala volume in women but not in men. These inconsistent results may be due to differences in participant characteristics. The Tost *et al*. (2010) study involved 212 participants (103 males and 109 females) and the Wang *et al*. (2014) study included 290 participants (136 males and 154 female). In addition, both studies collected data from mostly young adults in their 20s and 30s. Compared to these studies, our research has a larger number of participants (410; 211 males and 199 females) and a wider age range (ages 20 to 59). We believe that our approach contributes to an improved understanding of the relationship between genetic polymorphism, amygdala volume and attitudinal trust.

The cultural difference among GG genotype participants is another possible source for data discrepancies. Previously, cross-cultural studies found that the frequency of the GG genotype on *OXTR* rs53576 was very small in East Asian samples compared to American samples (Kim *et al*., 2010). Indeed, the number of GG genotypes in an East Asian sample was small [22 (7.6% of the sample) in Wang *et al*., (2014), 51 (12.4% of the sample) in our study] compared to the American sample [95 (45% of the sample) in Tost *et al*., (2010)]. Such a biased frequency of the GG genotype is thought to impair the consistency of the results between various studies.

We observed a difference in the association between *OXTR* rs53576 genotypes and amygdala volume among the sexes, which may be attributable to sex differences in the distribution of oxytocin receptors in the amygdala regulated by *OXTR* rs53576. We speculated that men with a GG genotype have more oxytocin receptors in the amygdala, whereas women with a AA genotype have more receptors; furthermore, it attenuated activation of the amygdala. Correspondingly, the amygdala volume decreased in men with GG genotype and women with AA genotype. However, the effects of *OXTR* rs53576 variations on oxytocin receptors are unknown and are currently difficult to examine *in vivo* in humans. Further studies and advances in technology to facilitate *in vivo* experiments are needed for a more comprehensive investigation into the relationship between the genetic variance of *OXTR* rs53576 and oxytocin receptors.

Second, we found a significant interaction effect between the left amygdala volume and sex on attitudinal trust measured in the first phase. In addition, the left amygdala volume was negatively associated with attitudinal trust in men, but not in women. Previous neuroscience studies have shown that the amygdala plays a pivotal role in the fear of risk during social exchange settings (Baumgartner *et al*., 2008; Koscik and Tranel, 2011). Tian *et al*. (2016) found that the left amygdala volume was positively correlated with social anxiety levels, and a recent meta-analysis reported that the left amygdala volume was positively associated with negative emotionality-related traits (Mincic, 2015). In addition, while the right amygdala plays a role in the automatic response that detects an emotional stimulus, the left amygdala plays a role in the cognitive process of emotion (Vrtička *et al*., 2011). Because attitudinal trust is an evaluation of the trustworthiness of others, the participants answered what they think about it in daily life. Therefore, men with small left amygdala volumes may exhibit higher levels of attitudinal trust and less fear of social risks, such as betrayal by others, compared to men with large left amygdala volumes. The association between attitudinal trust and the left amygdala volume was observed in men, but not in women, implying that the level of attitudinal trust in men is affected by fear of social risk mediated by amygdala volume. In women, factors other than the fear of social risk may have a greater effect on attitudinal trust levels.

The weaker relationship between attitudinal trust measured during the seventh phase compared to the first phase may be due to the time lapse between measurements. While the time between the first attitudinal trust assessment and MRI data collection is 6 months, the time between the second attitudinal trust assessment and MRI data collection is 2 years. These results suggest that the association between brain volume and psychological traits may become weaker with time.

Third, we revealed that the left amygdala volume has a significant mediation effect on the association between *OXTR* rs53576 genotypes and attitudinal trust in men. Nishina *et al*. (2015) found an association between *OXTR* rs53576 genotypes and attitudinal trust in men, although the pathway from gene to behaviour remained unclear. To our knowledge, this is the first study to show that left amygdala volume plays a pivotal role by mediating the relationship between *OXTR* rs53576 genotypes and attitudinal trust in men. Understanding the effects of *OXTR* rs53576 genotypes is important because they may be associated with the amount of oxytocin receptors in the amygdala. We hypothesised that more oxytocin receptors are present in the amygdala of GG men than in the amygdala of AG and AA men; the number of receptors will influence amygdala activation.

As many studies have shown that oxytocin administration attenuates amygdala activation (Kirsch *et al*., 2005; Baumgartner *et al*., 2008; Domes *et al*., 2007), oxytocin does not affect social cognition and social behaviour directly but affects the amygdala instead. Bartz *et al*. (2011) suggested the ‘anxiety reduction hypothesis’ as a model of the social effect on oxytocin. According to this hypothesis, oxytocin reduces anxiety and fear by acting on the amygdala, which regulates social cognition and social behaviour. This hypothesis is consistent with our results in this study that show that the oxytocin system reduces anxiety for social risk and regulates attitudinal trust.

Findings from studies on human trust with biological approaches are consistent with the findings of studies on human trust in the field of social sciences. As described previously, the frequency of the GG genotype in East Asians is lower than that in Americans, whereas the frequency of the AA genotype in East Asians is higher than that in Americans (Kim *et al*., 2010; Kim *et al*., 2011; Sasaki *et al*., 2011). In addition, the GG genotype is associated with high levels of attitudinal and behavioural trust in men (Krueger *et al*., 2012; Nishina et al., 2015). These results predict that the level of trust in American men is higher than that in East Asian men; this prediction is partially supported by studies on social psychology (Yamagishi and Yamagishi, 1994; Yamagishi, 2011). Yamagishi *et al*. (2011) discussed that high levels of trust in Americans are formed to adapt to the social environment, with a major advantage of increased interaction with strangers. This discussion suggests the possibility that *OXTR* and culture have coevolved.

### Limitations and future directions

We analysed the association among genotype, brain volume and attitudinal trust; however, attitudinal trust is a very limited aspect of human trust. In order to generalise these associations, it is necessary to consider various aspects of human trust (e.g. trust behaviour in daily life). Although we showed that the left amygdala volume plays a role in the association between *OXTR* rs53576 genotypes and attitudinal trust in men, future studies should examine whether the function of the left amygdala mediates this association as well. Furthermore, a few studies have reported that the level of DNA methylation in *OXTR* is associated with social anxiety (Ziegler *et al*., 2015), postpartum depression (Kimmel *et al*., 2016), and facial recognition ability (Puglia *et al*., 2015). Because DNA methylation is an indicator of gene expression (Saxonov *et al*., 2006; Moore *et al*., 2013), examining the relationship between methylation levels of *OXTR*, the left amygdala volume and attitudinal trust may enhance our understanding of the biological mechanism of trust.

## Supplementary Material

Supplementary Data

## References

[ref1] Apicella C.L., Cesarini D., Johannesson M., et al. (2010). No association between oxytocin receptor (*OXTR*) gene polymorphisms and experimentally elicited social preferences. PloS One, 5, e11153. 10.1371/journal.pone.0011153 PMC2886839 20585395

[ref2] Barber B. (1983). The Logic and Limits of Trust, New Brunswick, NJ: Rutgers University Press.

[ref3] Bartz J.A., Zaki J., Bolger N., Ochsner K.N. (2011). Social effects of oxytocin in humans: context and person matter. Trends in Cognitive Sciences, 15(7), 301–9. 21696997 10.1016/j.tics.2011.05.002

[ref4] Baumgartner T., Heinrichs M., Vonlanthen A., Fischbacher U., Fehr E. (2008). Oxytocin shapes the neural circuitry of trust and trust adaptation in humans. Neuron, 58, 639–50. 18498743 10.1016/j.neuron.2008.04.009

[ref5] Berg J., Dickhaut J., McCabe K. (1995). Trust, reciprocity, and social history. Games and Economic Behavior, 10, 122–42.

[ref6] Bohnet I., Zeckhauser R. (2004). Trust, risk and betrayal. Journal of Economic Behavior & Organization, 55, 467–84.

[ref7] Cesarini D., Dawes C.T., Fowler J.H., Johannesson M., Lichtenstein P., Wallace B. (2008). Heritability of cooperative behavior in the trust game. Proceedings of the National Academy of Sciences, 105(10), 3721–6. 10.1073/pnas.0710069105 PMC2268795 18316737

[ref8] Domes G., Heinrichs M., Gläscher J., Büchel C., Braus D.F., Herpertz S.C. (2007). Oxytocin attenuates amygdala responses to emotional faces regardless of valence. Biological Psychiatry, 62(10), 1187–90. 17617382 10.1016/j.biopsych.2007.03.025

[ref9] Donaldson Z.R., Young L.J. (2008). Oxytocin, vasopressin, and the neurogenetics of sociality. Science, 322, 900–4. 18988842 10.1126/science.1158668

[ref10] Febo M., Numan M., Ferris C.F. (2005). Functional magnetic resonance imaging shows oxytocin activates brain regions associated with mother–pup bonding during suckling. The Journal of Neuroscience, 25, 11637–44. 16354922 10.1523/JNEUROSCI.3604-05.2005 PMC6726012

[ref11] Inoue T., Kimura T., Azuma C., et al. (1994). Structural organization of the human oxytocin receptor gene. Journal of Biological Chemistry, 269, 32451–6. 7798245

[ref12] Insel T.R., Shapiro L.E. (1992). Oxytocin receptor distribution reflects social organization in monogamous and polygamous voles. Proceedings of the National Academy of Sciences, 89, 5981–5. 10.1073/pnas.89.13.5981 PMC402122 1321430

[ref13] Kanai R., Rees G. (2011). The structural basis of inter-individual differences in human behaviour and cognition. Nature Reviews Neuroscience, 12, 231–42. 21407245 10.1038/nrn3000

[ref14] Kim H.S., Sherman D.K., Mojaverian T., et al. (2011). Gene–culture interaction: oxytocin receptor polymorphism (*OXTR*) and emotion regulation. Social Psychological and Personality Science, 2, 665–72.

[ref15] Kim H.S., Sherman D.K., Sasaki J.Y., et al. (2010). Culture, distress, and oxytocin receptor polymorphism (*OXTR*) interact to influence emotional support seeking. Proceedings of the National Academy of Sciences of the United States of America, 107, 15717–21. 20724662 10.1073/pnas.1010830107 PMC2936623

[ref16] Kimmel M., Clive M., Gispen F., et al. (2016). Oxytocin receptor DNA methylation in postpartum depression. Psychoneuroendocrinology, 69, 150–60. 27108164 10.1016/j.psyneuen.2016.04.008 PMC7152506

[ref17] Kirsch P., Esslinger C., Chen Q., et al. (2005). Oxytocin modulates neural circuitry for social cognition and fear in humans. Journal of Neuroscience, 25(49), 11489–93. 16339042 10.1523/JNEUROSCI.3984-05.2005 PMC6725903

[ref18] Knack S., Keefer P. (1997). Does social capital have an economic payoff? A cross-country investigation. The Quarterly Journal of Economics, 112, 1251–88.

[ref19] Koscik T.R., Tranel D. (2011). The human amygdala is necessary for developing and expressing normal interpersonal trust. Neuropsychologia, 49, 602–11. 20920512 10.1016/j.neuropsychologia.2010.09.023 PMC3056169

[ref20] Kosfeld M., Heinrichs M., Zak P.J., Fischbacher U., Fehr E. (2005). Oxytocin increases trust in humans. Nature, 435, 673–6. 15931222 10.1038/nature03701

[ref21] Krueger F., Parasuraman R., Iyengar V., et al. (2012). Oxytocin receptor genetic variation promotes human trust behavior. Frontiers in Human Neuroscience, 6, 4. 22347177 10.3389/fnhum.2012.00004 PMC3270329

[ref22] Machado-de-Sousa J.P., Lima Osório F. de, Jackowski A.P., et al. (2014). Increased amygdalar and hippocampal volumes in young adults with social anxiety. PloS One, 9, e88523. 10.1371/journal.pone.0088523 PMC3921212 24523911

[ref23] Maldjian J.A., Laurienti P.J., Kraft R.A., Burdette J.H. (2003). An automated method for neuroanatomic and cytoarchitectonic atlas-based interrogation of fMRI data sets. Neuroimage, 19, 1233–9. 12880848 10.1016/s1053-8119(03)00169-1

[ref24] Matsumoto Y., Yamagishi T., Li Y., Kiyonari T. (2016). Prosocial behavior increases with age across five economic games. PloS One, 11, e0158671. 10.1371/journal.pone.0158671 PMC4945042 27414803

[ref25] Meyer-Lindenberg A., Domes G., Kirsch P., Heinrichs M. (2011). Oxytocin and vasopressin in the human brain: social neuropeptides for translational medicine. Nature Reviews Neuroscience, 12, 524–38. 21852800 10.1038/nrn3044

[ref26] Mincic A.M. (2015). Neuroanatomical correlates of negative emotionality-related traits: a systematic review and meta-analysis. Neuropsychologia, 77, 97–118. 26265397 10.1016/j.neuropsychologia.2015.08.007

[ref27] Moore L.D., Le T., Fan G. (2013). DNA methylation and its basic function. Neuropsychopharmacology, 38, 23–38. 22781841 10.1038/npp.2012.112 PMC3521964

[ref28] Nave G., Camerer C., McCullough M. (2015). Does oxytocin increase trust in humans? A critical review of research. Perspectives on Psychological Science, 10(6), 772–89. 26581735 10.1177/1745691615600138

[ref29] Nishina K., Takagishi H., Inoue-Murayama M., Takahashi H., Yamagishi T. (2015). Polymorphism of the oxytocin receptor gene modulates behavioral and attitudinal trust among men but not women. PloS One, 10, e0137089. 10.1371/journal.pone.0137089 PMC4621758 26444016

[ref30] Ophir A.G., Gessel A., Zheng D.J., Phelps S.M. (2012). Oxytocin receptor density is associated with male mating tactics and social monogamy. Hormones and Behavior, 61, 445–53. 22285648 10.1016/j.yhbeh.2012.01.007 PMC3312950

[ref31] Puglia M.H., Lillard T.S., Morris J.P., Connelly J.J. (2015). Epigenetic modification of the oxytocin receptor gene influences the perception of anger and fear in the human brain. Proceedings of the National Academy of Sciences, 112, 3308–13. 10.1073/pnas.1422096112 PMC4372000 25675509

[ref32] Putnam R.D. (1993). Making democracy work: civic traditions in modern Italy, Princeton: Princeton University Press.

[ref33] Sasaki J.Y., Kim H.S., Xu J. (2011). Religion and well-being: the moderating role of culture and the oxytocin receptor (*OXTR*) gene. Journal of Cross-Cultural Psychology, 42, 1394–405.

[ref34] Saxonov S., Berg P., Brutlag D.L. (2006). A genome-wide analysis of CpG dinucleotides in the human genome distinguishes two distinct classes of promoters. Proceedings of the National Academy of Sciences, 103, 1412–7. 10.1073/pnas.0510310103 PMC1345710 16432200

[ref35] Tian X., Hou X., Wang K., Wei D., Qiu J. (2016). Neuroanatomical correlates of individual differences in social anxiety in a non-clinical population. Social Neuroscience, 11, 424–37. 26442578 10.1080/17470919.2015.1091037

[ref36] Tillfors M., Furmark T., Marteinsdottir I., et al. (2001). Cerebral blood flow in subjects with social phobia during stressful speaking tasks: a PET study. American Journal of Psychiatry, 158, 1220–6. 11481154 10.1176/appi.ajp.158.8.1220

[ref37] Tillfors M., Furmark T., Marteinsdottir I., Fredrikson M. (2002). Cerebral blood flow during anticipation of public speaking in social phobia: a PET study. Biological Psychiatry, 52, 1113–9. 12460694 10.1016/s0006-3223(02)01396-3

[ref38] Tost H., Kolachana B., Hakimi S., et al. (2010). A common allele in the oxytocin receptor gene (*OXTR*) impacts prosocial temperament and human hypothalamic-limbic structure and function. Proceedings of the National Academy of Sciences, 107, 13936–41. 10.1073/pnas.1003296107 PMC2922278 20647384

[ref39] Uslaner E.M., Rothstein B. (2005). All for one: equality, corruption, and social trust. World Politics, 58, 41–72.

[ref40] Veenema A.H., Neumann I.D. (2008). Central vasopressin and oxytocin release: regulation of complex social behaviours. Progress in Brain Research, 170, 261–76. 18655888 10.1016/S0079-6123(08)00422-6

[ref41] Vrtička P., Sander D., Vuilleumier P. (2011). Effects of emotion regulation strategy on brain responses to the valence and social content of visual scenes. Neuropsychologia, 49, 1067–82. 21345342 10.1016/j.neuropsychologia.2011.02.020

[ref42] Wang J., Qin W., Liu B., et al. (2014). Neural mechanisms of oxytocin receptor gene mediating anxiety-related temperament. Brain Structure and Function, 219, 1543–54. 23708061 10.1007/s00429-013-0584-9

[ref43] Yamagishi T. (2011). *Trust: The Evolutionary Game of Mind and Society*, English edn, New York: Springer, Shinrai no kozo: Kokoroto shakaino shinnka geemu. Tokyo: Tokyo University Press. 1998.

[ref44] Yamagishi T., Akutsu S., Cho K., Inoue Y., Li Y., Matsumoto Y. (2015). Two-component model of general trust: predicting behavioral trust from attitudinal trust. Social Cognition, 33, 436.

[ref45] Yamagishi T., Li Y., Matsumoto Y., Kiyonari T. (2016a). Moral bargain hunters purchase moral righteousness when it is cheap: within-individual effect of stake size in economic games. Scientific Reports, 6, 27824. 10.1038/srep27824 PMC4906282 27296466

[ref46] Yamagishi T., Li Y., Takagishi H., Matsumoto Y., Kiyonari T. (2014). In search of Homo economicus. Psychological Science, 25, 1699–711. 25037961 10.1177/0956797614538065

[ref47] Yamagishi T., Takagishi H., Fermin A.D.S.R., Kanai R., Li Y., Matsumoto Y. (2016b). Cortical thickness of the dorsolateral prefrontal cortex predicts strategic choices in economic games. Proceedings of the National Academy of Sciences, 113, 5582–7. 10.1073/pnas.1523940113 PMC4878491 27140622

[ref48] Yamagishi T., Yamagishi M. (1994). Trust and commitment in the United States and Japan. Motivation and Emotion, 18, 129–66.

[ref49] Zhong S., Monakhov M., Mok H.P., et al. (2012). U-shaped relation between plasma oxytocin levels and behavior in the trust game. PLoS One, 7, e51095. 10.1371/journal.pone.0051095 PMC3515439 23227239

[ref50] Ziegler C., Dannlowski U., Bräuer D., et al. (2015). Oxytocin receptor gene methylation: converging multilevel evidence for a role in social anxiety. Neuropsychopharmacology, 40, 1528–38. 25563749 10.1038/npp.2015.2 PMC4397412

