# Supplementary material for: Association of the oxytocin receptor gene with attitudinal trust: role of amygdala volume
Source: Soc Cogn Affect Neurosci. 2018 Sep 7;13(10):1091–7. doi: 10.1093/scan/nsy075 (PMC6204480; doi:10.1093/scan/nsy075)
Supplement: Supplementary Data [file nsy075_supp.docx]

Supplementary Material

**Association of the oxytocin receptor gene with attitudinal trust in men:**

**role of the amygdala volume**

Kuniyuki Nishina, Haruto Takagishi, Fermin Alan, Miho Inoue-Murayama,

Hidehiko Takahashi, Masamichi Sakagami, Toshio Yamagishi


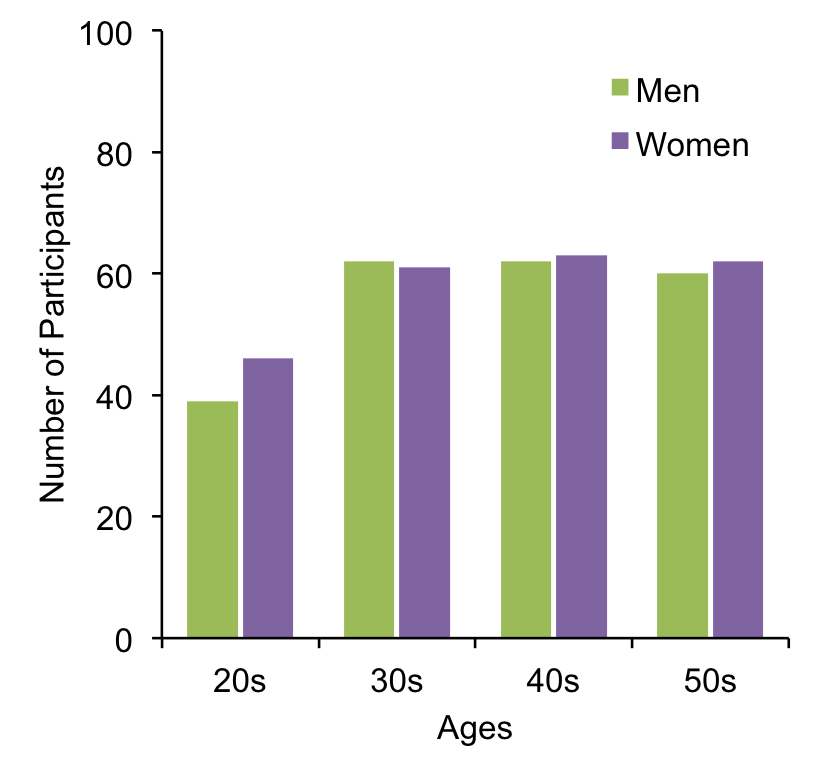
Figure S1. Participants’ age and gender

Figure S2. Participants’ education and gender
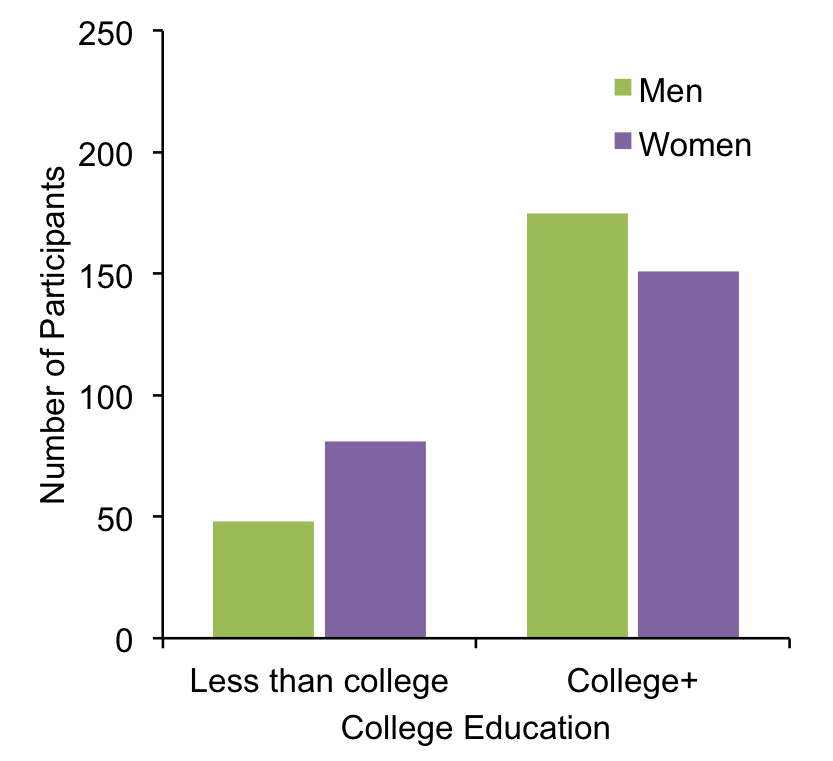


Figure S3. Participants’ annual income and gender
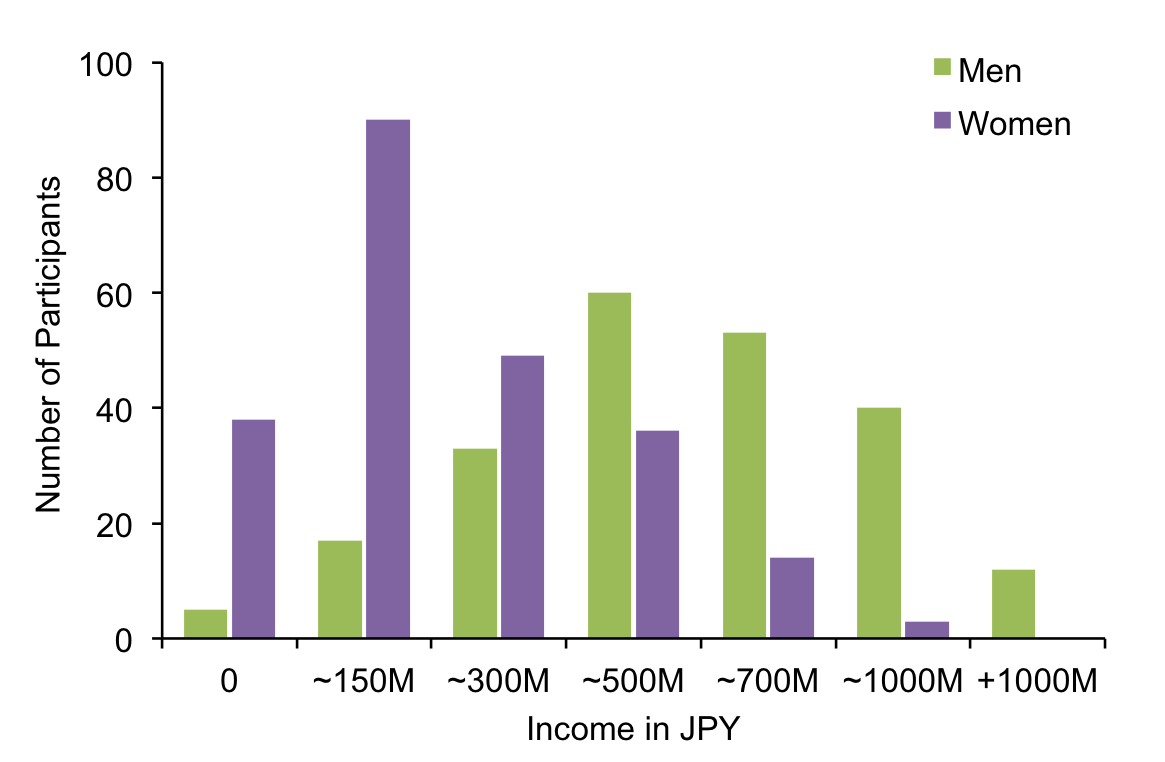


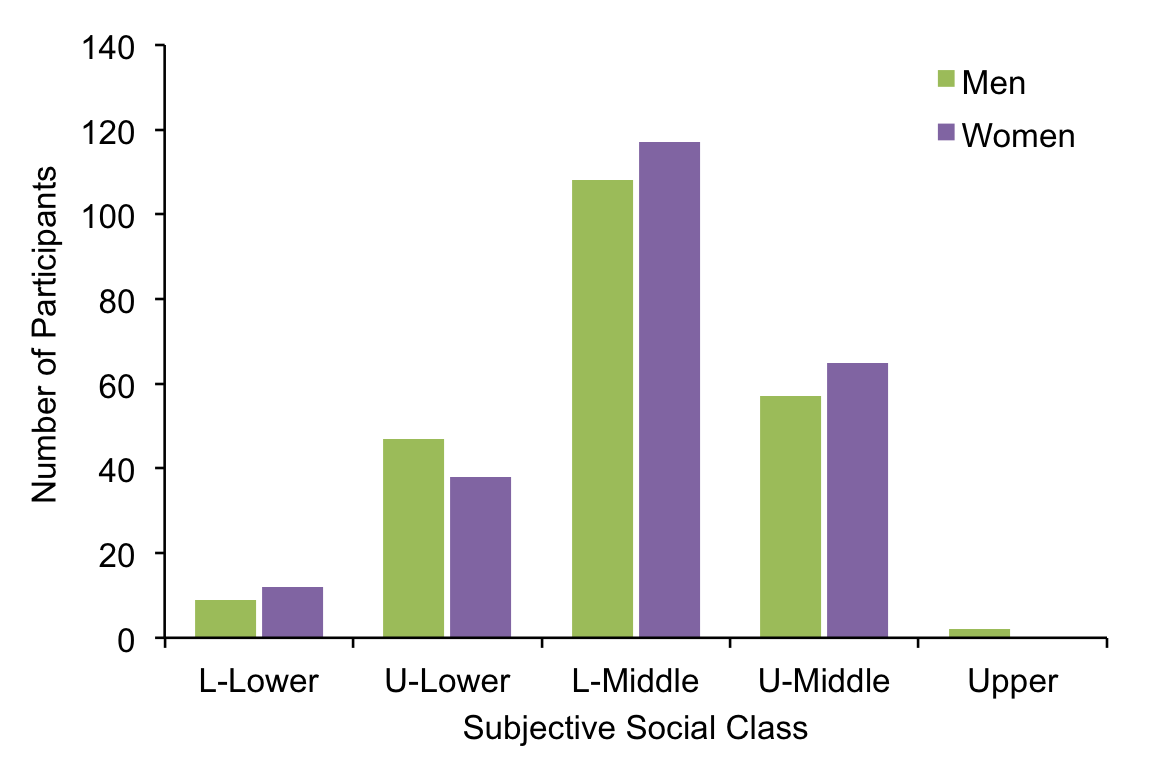
Figure S4. Participants’ subjective social class and gender


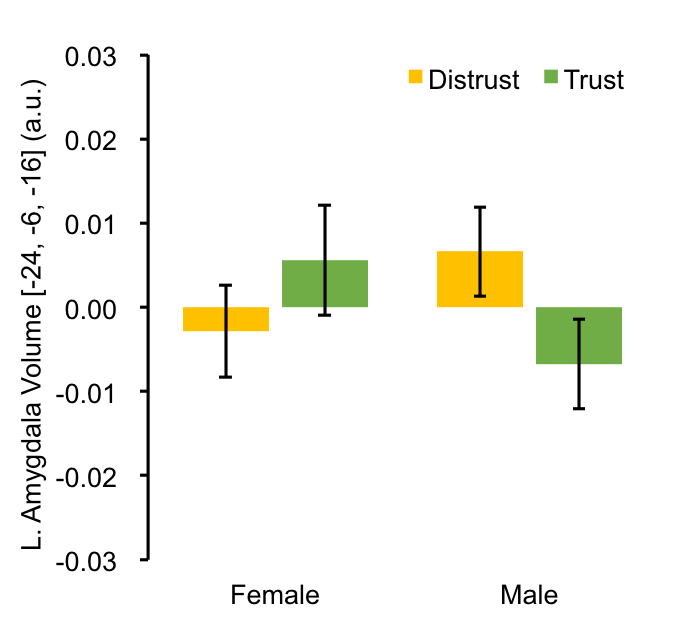


Figure S5. Mean volume of the left amygdala according to the participants’ levels of attitudinal trust measured in the seventh phase by sex. Participants who answered NO to the question were assigned to the distrust group, while those who answered YES were assigned to the trust group. Error bars show standard errors.

| Table S1 Demographics and genotype distribution by sex and generation | | | | | | | | | | | | | |
| --- | --- | --- | --- | --- | --- | --- | --- | --- | --- | --- | --- | --- | --- |
|  |  | 20s | | | 30s | | | 40s | | | 50s | | |
| Sex | Education | AA | AG | GG | AA | AG | GG | AA | AG | GG | AA | AG | GG |
| Men | Less than college | 5 | 2 | 1 | 5 | 4 | 4 | 9 | 4 | 3 | 3 | 4 | 0 |
|  | College+ | 12 | 15 | 3 | 22 | 20 | 6 | 15 | 20 | 5 | 19 | 22 | 8 |
| Women | Less than college | 3 | 6 | 0 | 7 | 13 | 0 | 8 | 14 | 1 | 8 | 10 | 2 |
|  | College+ | 8 | 17 | 6 | 15 | 15 | 3 | 13 | 13 | 3 | 14 | 14 | 6 |

| Table S2. Annual income and genotype distribution by sex and generation | | | | | | | | | | | | | |
| --- | --- | --- | --- | --- | --- | --- | --- | --- | --- | --- | --- | --- | --- |
|  |  | 20s | | | 30s | | | 40s | | | 50s | | |
| Sex | Annual income | AA | AG | GG | AA | AG | GG | AA | AG | GG | AA | AG | GG |
| Men | 0 | 1 | 0 | 0 | 0 | 0 | 1 | 1 | 1 | 0 | 0 | 0 | 0 |
|  | ~150M | 3 | 4 | 1 | 1 | 3 | 0 | 0 | 0 | 2 | 0 | 3 | 0 |
|  | ~300M | 1 | 1 | 2 | 8 | 5 | 2 | 3 | 3 | 2 | 4 | 0 | 0 |
|  | ~500M | 8 | 11 | 1 | 10 | 6 | 4 | 5 | 4 | 2 | 3 | 3 | 0 |
|  | ~700M | 1 | 1 | 0 | 6 | 7 | 1 | 10 | 12 | 1 | 4 | 8 | 1 |
|  | ~1000M | 0 | 0 | 0 | 2 | 3 | 2 | 4 | 3 | 0 | 9 | 7 | 5 |
|  | +1000M | 0 | 0 | 0 | 0 | 0 | 0 | 1 | 1 | 1 | 2 | 5 | 2 |
| Women | 0 | 1 | 7 | 0 | 3 | 4 | 1 | 5 | 6 | 0 | 4 | 2 | 2 |
|  | ~150M | 5 | 7 | 2 | 6 | 10 | 0 | 10 | 11 | 2 | 10 | 11 | 4 |
|  | ~300M | 3 | 5 | 3 | 8 | 8 | 0 | 3 | 2 | 0 | 3 | 5 | 0 |
|  | ~500M | 2 | 3 | 1 | 3 | 5 | 2 | 2 | 5 | 1 | 2 | 4 | 1 |
|  | ~700M | 0 | 0 | 0 | 2 | 1 | 0 | 0 | 3 | 1 | 2 | 1 | 1 |
|  | ~1000M | 0 | 0 | 0 | 0 | 0 | 0 | 1 | 0 | 0 | 1 | 0 | 0 |
|  | +1000M | 0 | 0 | 0 | 0 | 0 | 0 | 0 | 0 | 0 | 0 | 0 | 0 |

| Table S3. Subjective social class and genotype distribution by sex and generation | | | | | | | | | | | | | |
| --- | --- | --- | --- | --- | --- | --- | --- | --- | --- | --- | --- | --- | --- |
|  |  | 20s | | | 30s | | | 40s | | | 50s | | |
| Sex | Subjective social class | AA | AG | GG | AA | AG | GG | AA | AG | GG | AA | AG | GG |
| Men | L-Lower | 0 | 1 | 0 | 0 | 2 | 1 | 1 | 1 | 0 | 1 | 1 | 0 |
|  | U-lower | 5 | 5 | 2 | 9 | 5 | 2 | 6 | 7 | 2 | 1 | 1 | 0 |
|  | L-Middle | 6 | 7 | 2 | 13 | 10 | 6 | 13 | 12 | 5 | 9 | 16 | 3 |
|  | U-Middle | 6 | 4 | 0 | 5 | 7 | 1 | 3 | 4 | 1 | 11 | 8 | 4 |
|  | Upper | 0 | 0 | 0 | 0 | 0 | 0 | 1 | 0 | 0 | 0 | 0 | 1 |
| Women | L-Lower | 3 | 2 | 1 | 0 | 2 | 0 | 0 | 1 | 0 | 0 | 0 | 0 |
|  | U-lower | 3 | 4 | 1 | 5 | 3 | 0 | 4 | 4 | 0 | 4 | 5 | 0 |
|  | L-Middle | 4 | 13 | 3 | 12 | 14 | 3 | 11 | 13 | 3 | 11 | 12 | 2 |
|  | U-Middle | 1 | 4 | 1 | 5 | 9 | 0 | 6 | 9 | 1 | 7 | 7 | 6 |
|  | Upper | 0 | 0 | 0 | 0 | 0 | 0 | 0 | 0 | 0 | 0 | 0 | 0 |
